# The Impact of Visual Feedback Design on Self-Regulation Performance and Learning in a Single-Session rt-fMRI Neurofeedback Study at 3T and 7T

**DOI:** 10.3390/brainsci16020166

**Published:** 2026-01-30

**Authors:** Sebastian Baecke, Ralf Lützkendorf, Johannes Bernarding

**Affiliations:** Institute of Medical Data Science, Otto-von-Guericke University, 39120 Magdeburg, Germany; ralf.luetzkendorf@med.ovgu.de (R.L.); johannes.bernarding@med.ovgu.de (J.B.)

**Keywords:** real-time fMRI, neurofeedback, sensorimotor cortex, self-regulation, feedback modality, continuous visual feedback, categorical (affective) feedback

## Abstract

**Background:** The efficacy of real-time fMRI neurofeedback (NFB) depends critically on how feedback is presented and perceived by the participant. Although various visual feedback designs are used in practice, there is limited evidence on the impact of modality on learning and performance. We conducted a feasibility study to compare the effectiveness of different feedback modalities, and to evaluate the technical performance of NFB across two scanner field strengths. **Methods:** In a single-session study, nine healthy adults (6 men, 3 women) voluntarily adapted the activation level of the primary sensorimotor cortex (SMC) to reach three predefined activation levels. We contrasted a continuous, signal-proportional feedback (cFB; a thermometer-style bar) with an affect-based, categorical feedback (aFB; a smiling face). A no-feedback transfer condition (noFB) was included to probe regulation based on internal representations alone. To assess technical feasibility, three participants were scanned at 7T and six at 3T. **Results:** Participants achieved successful regulation in 44.4% of trials overall (cFB 46.9%, aFB 43.8%, noFB 42.6%). Overall success rates did not differ significantly between modalities and field strengths when averaged across the session; given the small feasibility sample, this null result is inconclusive and does not establish equivalence. Learning effects were modality-specific. Only cFB showed a significant within-session improvement (+14.8 percentage points from RUN1 to RUN2; *p* = 0.031; d_z = 0.94), whereas aFB and noFB showed no evidence of learning. Exploratory whole-brain contrasts (uncorrected) suggested increased recruitment of ipsilateral motor regions during noFB. The real-time pipeline demonstrated robust technical performance: transfer/reconstruction latency averaged 497.8 ms and workstation processing averaged 296.8 ms (≈795 ms end-to-end), with rare stochastic outliers occurring predominantly during 7T sessions. **Conclusions:** In this single-session motor rt-fMRI NFB paradigm, continuous signal-proportional feedback supported rapid within-session learning, whereas affect-based categorical cues did not yield comparable learning benefits. Stable low-latency operation was achievable at both 3T and 7T. Larger, balanced studies are warranted to confirm modality-by-learning effects and to better characterize transfer to feedback-free self-regulation.

## 1. Introduction

Neurofeedback (NFB) is a form of biofeedback that operates within a closed-loop paradigm, enabling individuals to learn volitional control over their own neural activity [1]. This is achieved by providing real-time information about a specific brain signal that is otherwise inaccessible to conscious awareness [1,2,3]. While NFB based on electroencephalography (EEG) has a long and successful history in clinical applications [3], particularly for treating conditions such as attention-deficit/hyperactivity disorder [4,5,6], it is limited by its spatial resolution [7]. In contrast, real-time functional magnetic resonance imaging (rt-fMRI) offers superior spatial specificity and whole-brain coverage. This technological advance allows for the non-invasive targeting of specific cortical regions and deep subcortical structures, such as the amygdala or insula, opening new avenues for neurorehabilitation and psychiatric intervention [8,9,10]. However, despite this promise, recent systematic and meta-analytic work indicates that effect sizes of rt-fMRI NFB interventions are often modest and heterogeneous, highlighting the need to better understand which design choices most strongly influence learning and outcome.

In a typical rt-fMRI NFB setup, the blood-oxygenation-level-dependent (BOLD) signal from a predefined region of interest (ROI) serves as an indirect proxy for neuronal activity [7,8]. This signal is processed in real time and presented back to the participant, usually via a visual or auditory display [8]. The participant’s task is to modulate this signal towards a predefined target by employing specific mental strategies, such as motor imagery, emotion regulation, or focused attention [1]. Across repeated attempts, successful regulation is thought to reinforce task-relevant neural pathways and to update internal forward models of action, leading to lasting changes in brain–behavior relationships through principles of operant conditioning and motor skill learning [1,11,12,13].

A critical, yet often under-investigated, component of this learning process is the design of the feedback display itself. The way information is presented can profoundly influence a participant’s ability to learn and perform the task, embodying a fundamental trade-off between informational precision and motivational salience in rt-fMRI NFB design [2,14]. On one hand, feedback can be designed for maximal precision and control. This approach typically employs abstract, quantitative displays, such as a simple thermometer-style bar, which provide a continuous and proportional representation of the neural signal [8,9]. The high granularity of this information directly answers the question “What is my exact signal value and how close am I to the target?”. By minimizing the need for interpretation, such displays reduce cognitive load and allow the participant to directly and quantitatively assess the efficacy of their mental strategy. This direct mapping is hypothesized to facilitate fine-grained adjustments and a strong sense of control, potentially accelerating the learning process [15]. However, a potential disadvantage is that such displays may be perceived as less engaging, more “technical,” or even boring, which could impact motivation over time.

On the other hand, feedback can be designed to enhance motivation and engagement. This is often achieved through more complex, emotionally or socially salient cues, such as a smiling face [16]. This approach typically relies on categorical scaling, where neural activity is translated into discrete levels (e.g., a neutral expression, a slight smile, a broad smile) [2]. While this may be more motivating through social reward mechanisms [17], it comes at the cost of informational precision. Recent studies using such naturalistic face feedback for amygdala regulation, for instance, have shown mixed success, suggesting that factors such as emotional valence and task congruency introduce a layer of complexity that can affect learning outcomes [18]. Such feedback offers low granularity, answering only “Which level am I in?” but providing no information about the precise position within that level or the proximity to the next threshold. This can lead to ambiguity and frustration, as small, meaningful signal fluctuations remain invisible, while crossing a threshold results in an abrupt change in the displayed stimulus. Furthermore, the need to decode a social cue introduces an additional cognitive demand that may distract from the primary task of self-regulation [19].

While both design philosophies are prevalent, there is a lack of consensus on which is more effective for rapid skill acquisition. Large meta- and mega-analytic efforts have further shown that predicting who will become a successful neurofeedback learner is non-trivial: pre-training activity in the target ROI explains at best a very small fraction of the variance in later learning, and performance in pre-training no-feedback runs does not reliably predict subsequent neurofeedback success [20]. More recent machine-learning work across 28 real-time fMRI neurofeedback experiments achieved only modest classification accuracy when predicting neurofeedback performance and improvement from a broad set of subject-, ROI- and design-level features, but identified several paradigm characteristics that are systematically associated with better performance, including the presence of a pre-training no-feedback run and the use of patient populations [21]. Together, this line of work suggests that optimizing modifiable aspects of the neurofeedback paradigm itself—such as the feedback display and other core protocol elements (e.g., the inclusion and structure of transfer runs)—may be a more promising route to improve learning success across diverse participants than attempting to select “good learners” based on baseline predictors. Complementing these design- and prediction-oriented findings, a systematic review of rt-fMRI NFB studies in clinical populations has highlighted substantial heterogeneity of protocols, small sample sizes and incomplete reporting of regulation and clinical outcomes, underlining the importance of well-characterized, method-focused feasibility work such as the present study [22].

Furthermore, the ultimate goal of any successful NFB intervention is the generalization of the learned self-regulation skill, allowing it to be applied in daily life without the need for an external feedback signal [1]. To bridge this gap, many NFB protocols incorporate “transfer runs” in which feedback is withheld [23]. These runs are essential for assessing whether a participant has developed a stable internal model of self-regulation [11], relying on interoceptive and proprioceptive cues [24], rather than simply learning to control a visual stimulus. The inclusion of such a no-feedback condition is therefore vital for a comprehensive evaluation of NFB learning [14,25].

In the present single-session feasibility study, we therefore asked how feedback design and the inclusion of a transfer condition influence self-regulation performance and learning in a simple motor rt-fMRI NFB paradigm. We used overt left-hand finger tapping to drive activity in a participant-specific region of interest in the right primary sensorimotor cortex centered on the hand area of the primary motor cortex (M1 ROI) [26,27]. Within this well-established model system, we directly contrasted three conditions: (i) continuous, signal-proportional feedback (cFB; thermometer-style bar) designed for maximal perceptual clarity; (ii) affect-based, categorical feedback (aFB; schematic smiling face) designed to provide a socially salient reward cue; and (iii) a no-feedback transfer condition (noFB) in which participants relied solely on internal sensory and motor cues while outcome information was provided only at block end. In addition, leveraging an existing multi-field-strength infrastructure, we evaluated the technical feasibility of implementing the same rt-fMRI NFB pipeline at 3T and 7T by quantifying end-to-end system latency and its variability. We hypothesized that continuous feedback would support faster within-session learning of M1 regulation than affect-based feedback or no-feedback transfer, and that the feasibility of the paradigm would generalize across field strengths despite differences in signal-to-noise ratio and data transfer demands.

## 2. Materials and Methods

### 2.1. Participants

Nine healthy, right-handed adults (6 males, 3 females; age: 28.0 ± 4.9 years) with normal or corrected-to-normal vision participated in the study. The study was approved by the Ethics Committee of the Otto-von-Guericke University Magdeburg, Faculty of Medicine. All participants provided written informed consent prior to participation in accordance with the Declaration of Helsinki and received financial compensation. Eligibility criteria included being at least 18 years old and having no history of neurological or psychiatric disorders or contraindications for MRI. No participants were excluded. In this exploratory feasibility study, participants were non-randomly assigned to the 3T (*n* = 6) or 7T (*n* = 3) scanner based on availability. This study was designed as an exploratory feasibility study to assess the viability of the paradigm and generate initial hypotheses. Consequently, a formal a priori power calculation was not performed, and the sample size was determined by practical constraints.

### 2.2. MRI Hardware and Image Acquisition

MRI data were acquired in a between-subjects design. Six participants were scanned on a 3T Siemens MAGNETOM Prisma (syngo MR VD13D, Siemens Healthineers, Erlangen, Germany), and three on a 7T Siemens whole-body investigational scanner (syngo MR VB17, Siemens Healthineers, Erlangen, Germany). For all participants, an identical high-resolution T1-weighted MPRAGE anatomical scan was acquired (voxel size = 0.8 × 0.8 × 0.8 mm^3^).

Functional images were collected using T2*-weighted gradient-echo EPI sequences. At 3T, a 64-channel head coil was used with the following parameters: TR/TE = 2000/30 ms; flip angle = 90°; 34 interleaved slices; voxel size = 2 × 2 × 3 mm^3^; 10% slice gap; GRAPPA factor = 3; bandwidth = 1785 Hz/px. At 7T, a 32-channel head coil was used, and the functional sequence was adapted as follows: TR/TE = 2000/22 ms; flip angle = 90°; 40 interleaved slices; voxel size = 2 × 2 × 3 mm^3^; no slice gap; GRAPPA factor = 4; bandwidth = 1515 Hz/px. For all functional scans, the phase-encoding direction was anterior-to-posterior.

### 2.3. ROI Localization

An individually defined functional region of interest (ROI) in the right primary sensorimotor cortex centered on the hand area of the primary motor cortex (M1) was obtained from a block-design finger-tapping localizer scan preceding the neurofeedback runs [28]. During this localizer (≈5:40 min total), participants performed overt left-hand finger tapping in ten 10 s task blocks (5 TRs) alternating with 20 s fixation baselines (10 TRs). An online general linear model (GLM) was computed continuously to generate an activation map contrasting tapping versus fixation in real time [29]. For each participant, the functional ROI used for real-time feedback was defined by applying a voxel-wise statistical threshold to the online GLM map and selecting the most significant activation cluster located anatomically within the precentral gyrus, corresponding to the primary motor hand area [30]. The statistical threshold was adjusted interactively for each participant (typically t = 4.0–7.0) to yield a compact, contiguous cluster; resulting ROI sizes varied across participants (10.1 ± 3.8 cm^3^). ROI definition was performed online in native functional space within TBV. This semi-automated procedure ensured that the ROI was centered on the core of the task-related activation while minimizing the inclusion of noisy or peripheral voxels, thereby optimizing the signal-to-noise ratio for the subsequent neurofeedback runs. The resulting activation mask was stored and used as the subject-specific right primary sensorimotor cortex ROI centered on M1 (hereafter referred to as the M1 ROI) for both online feedback computation and subsequent ROI-based offline analyses.

### 2.4. Real-Time Data Processing

Real-time data processing and feedback presentation were managed by Turbo-BrainVoyager (TBV) 3.2 (Brain Innovation, Maastricht, The Netherlands) using custom C++ plug-ins [31]. The online preprocessing pipeline included incremental 3D motion correction and online linear detrending; no physiology-based noise correction (e.g., RETROICOR/cardiac or respiratory regressors) was applied. System latency was quantified for each volume and is reported in detail in Section 3.

### 2.5. Neurofeedback Paradigm

**Figure 1:**
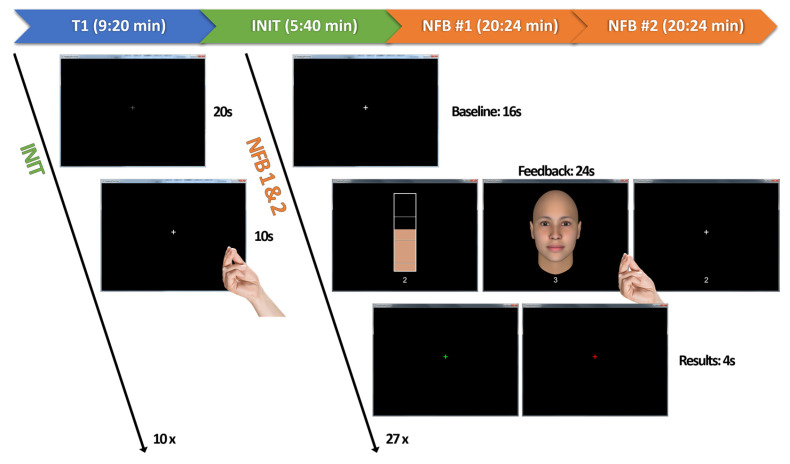
Session timeline and real-time fMRI (rt-fMRI) neurofeedback task design. The session began with a high-resolution T1-weighted anatomical scan, followed by a functional localizer (INIT) to define a participant-specific region of interest (ROI). Participants then completed two consecutive neurofeedback runs (NFB #1 and NFB #2). Each run consisted of 27 blocks, with each block (trial) comprising three phases: (1) an 8-TR (16 s) baseline period, (2) a 12-TR (24 s) regulation period, during which participants performed self-regulation operationalized as overt left-hand finger tapping at variable speed and force, and (3) a 2-TR (4 s) outcome cue. At the onset of the regulation period, a numeric cue (1–3) indicated the target activation level (weak, medium, or strong). Feedback was provided in one of three randomized modalities: continuous signal-proportional feedback (cFB; a thermometer-style bar), affect-based categorical feedback (aFB; a smiling face), or no-feedback (noFB; a static fixation cross without performance feedback). The block concluded with an outcome cue, where a green or red fixation cross indicated success or failure, respectively. Success for a given block was defined as reaching the target level for at least 50% of the regulation TRs. A white fixation cross was displayed during baseline periods.

The overall session timeline is depicted in Figure 1. Participants completed two consecutive neurofeedback runs (NFB #1 and NFB #2) in a single session, each lasting approximately 20:24 min. Each run began with an 8-TR (16 s) stabilization baseline and then comprised 27 blocks in a randomized order without replacement.

**Figure 2:**
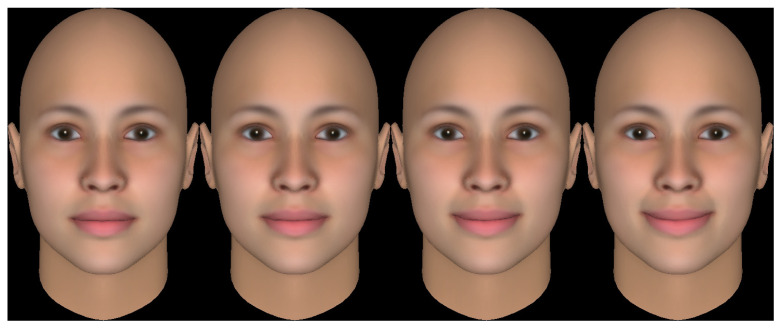
Affect-based categorical feedback (aFB) stimuli. The four pre-defined stimuli used in the aFB condition are displayed. The faces correspond to different levels of the normalized feedback signal S(t): The neutral face (leftmost) served as the baseline reference (shown during rest periods). A slight smile represented Level 1 (weak; 10% ≤ S(t) < 40%), a moderate smile represented Level 2 (medium; 40% ≤ S(t) < 70%), and a pronounced smile represented Level 3 (strong; S(t) ≥ 70%). To control for confounding variables, a gender-neutral European average face (FaceGen Artist Pro, Singular Inversions) was used, and parameters such as head pose, lighting, and background were held constant across all stimuli. During the regulation period of an aFB block, the displayed face updated once per TR to reflect the participant’s current brain activity level.

Each block followed a consistent structure, as illustrated in Figure 1. The block began with an 8-TR (16 s) baseline period. The subsequent regulation period was initiated by a numeric cue (‘1’, ‘2’, or ‘3’) indicating the required target activation level (weak, medium, or strong). During the 12-TR (24 s) regulation period, participants attempted to modulate activity in the M1 ROI by performing overt left-hand finger tapping while receiving visual feedback according to one of three randomly presented conditions. They were instructed to adjust the speed and perceived force of the finger movements to reach the cued target level (weak, medium, strong), while keeping movements confined to the left hand and minimizing arm and head motion:

(a) Continuous Signal-Proportional Feedback (cFB): The feedback signal was rendered as a vertical thermometer-style bar that updated once per TR. The bar’s height was directly proportional to the neural activity on a 0–100% scale.
(b) Affect-Based Categorical Feedback (aFB): The feedback signal was quantized into discrete levels and rendered as one of four pre-defined schematic faces, ranging from neutral to a pronounced smile (Figure 2), updating once per TR, in analogy to prior work using facial expressions as social reward cues [32]. The stimuli were designed to be gender-neutral and to convey positive affect only.
(c) No-Feedback (noFB): No performance-contingent feedback was provided. Participants were instructed to continue the learned left-hand finger tapping and to adjust movement speed and effort based on their internal sense of performance while viewing a static fixation cross identical to the one shown during baseline. This condition served as a transfer test [23], requiring participants to rely solely on their internal representations and strategies for regulating the M1 ROI via overt movement, in the absence of visual performance cues.

Each block concluded with a 2-TR (4 s) outcome phase. A green fixation cross indicated a “successful” block, whereas a red cross indicated “failure”. For all analyses, a block was classified as successful if the feedback signal met or exceeded the cued target level during at least 50% of the 12 regulation TRs (i.e., ≥ 6 TRs). This a priori threshold was chosen to balance sensitivity to partial target attainment against robustness to brief fluctuations in the BOLD signal.

The neurofeedback signal, denoted as S(t), was derived for each TR from the mean percent signal change (%∆BOLD) within the predefined ROI, relative to the mean of the immediately preceding baseline block [8,9]. For calibration, each participant’s maximum activation level from the localizer run was set to 100% on an internal display scale. Values exceeding 100% were clipped. Three target levels were defined a priori and applied identically across all modalities: Level 1 (weak), 10% ≤ S(t) < 40%; Level 2 (medium), 40% ≤ S(t) < 70%; and Level 3 (strong), S(t) ≥ 70%. Participant-specific scaling to the localizer maximum was chosen to harmonize feedback levels across individuals and across 3T and 7T sessions, where absolute %ΔBOLD amplitudes are expected to differ. While Level 3 was defined as an open-ended threshold (S(t) ≥ 70%), the continuous display scale was bounded to 0–100%, and values above 100% were clipped.

### 2.6. Offline Analyses

Offline Analyses of fMRI data were performed using the CONN toolbox [33] (RRID:SCR_009550) release 22.a and SPM (RRID:SCR_007037) release 12.7771 [34]. Functional and anatomical data were preprocessed using a modular preprocessing pipeline [35] including realignment with correction of susceptibility distortion interactions, slice timing correction, outlier detection, direct segmentation and MNI-space normalization, and smoothing.

Functional data were realigned using SPM realign & unwarp procedure, where all scans were coregistered to a reference image (first scan of the first session) using a least squares approach and a 6 parameter (rigid body) transformation, and resampled using b-spline interpolation to correct for motion and magnetic susceptibility interactions. Temporal misalignment between different slices of the functional data was corrected following SPM slice-timing correction (STC) procedure, using sinc temporal interpolation to resample each slice BOLD timeseries to a common mid-acquisition time. Potential outlier scans were identified using ART as acquisitions with framewise displacement above 0.9 mm or global BOLD signal changes above 5 standard deviations [36], and a reference BOLD image was computed for each subject by averaging all scans excluding outliers.

Functional and anatomical data were normalized into standard MNI space, segmented into gray matter, white matter, and CSF tissue classes, and resampled to 2 mm isotropic voxels following a direct normalization procedure [37] using SPM unified segmentation and normalization algorithm [38] with the default IXI-549 tissue probability map template. Last, functional data were smoothed using spatial convolution with a Gaussian kernel of 6 mm full width half maximum (FWHM). This preprocessing pipeline was designed to align with current best-practice recommendations for fMRI data analysis and reporting [39].

### 2.7. Statistical Analysis

Statistical analyses were performed using IBM SPSS Statistics (Version 29, GENLINMIXED procedure; IBM Corp., Armonk, NY, USA) and SAS software (SAS/STAT^®^ 9.2; SAS Institute Inc., Cary, NC, USA). To test the primary hypothesis that neurofeedback success probability depends on feedback type, we used a Generalized Linear Mixed Model (GLMM) [40] with binomial distribution (18 trials per condition) and logit link function. Fixed effects included feedback type (aFB, cFB, noFB), with data aggregated across runs. Within-subject correlation was modeled using heterogeneous compound symmetry covariance structure (selected via AICC: HCS = 44.9 vs. UN = 51.5, CS = 48.8), appropriate for the small sample (*n* = 9). To confirm robustness of our results, a non-parametric Friedman’s test was conducted as a complementary analysis. A significance level of α = 0.05 was used for all inferential tests. In addition, we conducted a sensitivity analysis using continuous, task-congruent performance metrics to mitigate potential information loss due to dichotomization. Specifically, we computed (i) occupancy, defined as the proportion of regulation TRs in which the feedback value fell within the instructed target range (0–1), and (ii) distance-to-range, defined per TR as 0 if the feedback value was within the target range and otherwise as the absolute distance to the nearest range boundary. Distance-to-range was summarized per block using its median (precision) and standard deviation (stability). These continuous metrics were aggregated at the participant level for each feedback type and run and compared between RUN1 and RUN2 using paired, two-sided Wilcoxon signed-rank tests (*n* = 9). A significance level of α = 0.05 was used for all inferential tests. Where applicable, the design, analysis, and reporting of this feasibility study were informed by current consensus recommendations for neurofeedback (CRED-nf) [25] and MRI data analysis and sharing (COBIDAS-MRI) [39].

## 3. Results

### 3.1. System Latency Analysis

We quantified real-time system performance by separating latency into two components: (i) transfer/reconstruction latency—the time from image acquisition to arrival of the reconstructed volume at the neurofeedback workstation; and (ii) computational processing time—the per-volume time required on the workstation to complete real-time preprocessing and statistical calculations, compute the neurofeedback value, and render the visual stimulus (e.g., thermometer bar, face display). Unless stated otherwise, latencies are reported in milliseconds (ms).

### 3.2. Image Transfer and Reconstruction Latency

**Figure 3:**
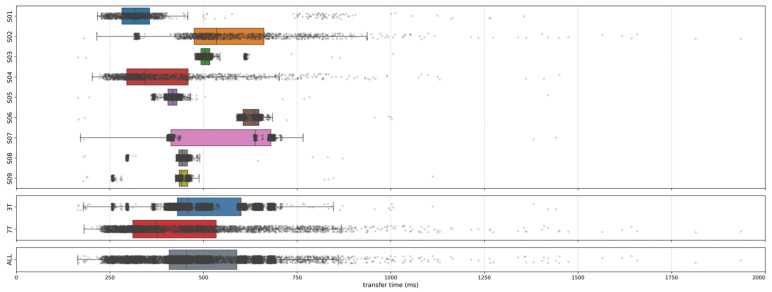
Transfer/reconstruction latency distributions across participants and field strengths. Top: participant-wise boxplots (S01–S09); S01, S02, S04 were scanned at 7T, the remainder at 3T. Middle: data aggregated by field strength (3T vs. 7T). Bottom: all volumes combined. Boxplots show medians (center line), IQR (box), and whiskers extending to 1.5 × IQR; points beyond whiskers are plotted as outliers (semi-transparent, jittered). The *x*-axis is truncated at 2000 ms for legibility; 44 volumes (0.36%) above this threshold (all 7T; 41 from S02) are not shown but are included in all summary statistics.

Across 12,276 volumes, transfer/reconstruction latency had a mean of 497.78 ms (SD 395.45; 99% CI [488.52, 507.04]) and was right-skewed with a median of 454 ms (IQR 181 ms; range 164–12,004 ms). Participant-wise boxplots revealed marked heterogeneity in central tendency and dispersion (Figure 3, top panel). For example, participant S02 exhibited higher median latency and greater variability relative to the more stable profile of S03. Aggregating by MRI field strength (Figure 3, middle panel; 3T: [S03, S05–S09]; 7T: [S01, S02, S04]) showed right-skewed distributions for both scanners; median latencies were comparable, with 7T displaying a descriptively broader spread. For visual clarity, the *x*-axis in Figure 3 is truncated at 2000 ms. This truncation masks 44 volumes (0.36%), all of which were acquired during 7T sessions. Of these, 41 were obtained from a single participant (S02). Of these, 22 occurred during regulation blocks (including three in noFB blocks with no feedback displayed), while the remaining 19 feedback-on events were confined to two consecutive aFB blocks (RUN1, S02). During these events, the display initially held the last valid state. All volumes, including these extremes, were included in the summary statistics; the truncation only affects visualization.

### 3.3. Computational Processing Time

Computational processing on the NFB workstation (Intel^®^ Core™ i7-3770, 3.40 GHz; 32 GB RAM) yielded a mean of 296.83 ms (SD 72.87; 99% CI [295.13, 298.54]), with a median of 300 ms (IQR 99 ms; Q1 = 260 ms, Q3 = 359 ms; range 5–478 ms) across the same 12,276 volumes. By definition, this metric includes real-time preprocessing, statistical calculations, neurofeedback value computation, and on-screen rendering of the visual stimulus.

### 3.4. Localizer-Based Determination of the Individual Target ROI and BOLD Response

**Figure 4:**
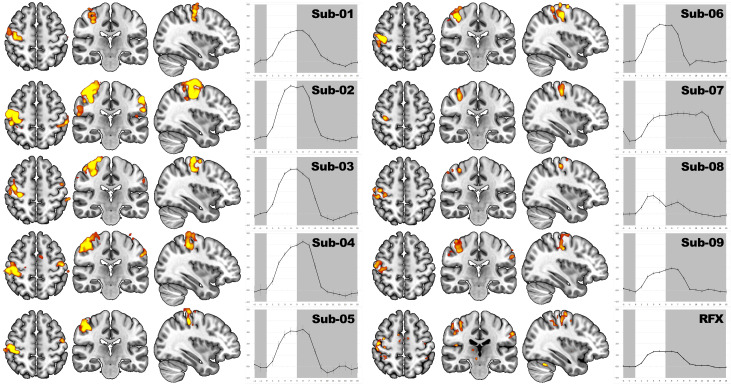
Localizer activation and subject-specific ROI time courses prior to the feedback measurement (INIT phase). For each participant, axial, coronal, and sagittal slices display significantly activated voxels on the ICBM MNI152 template at voxel-wise FWE-corrected *p* < 0.001 with minimum cluster size k ≥ 15. A group-level RFX map is shown at *p* < 0.005 uncorrected (k ≥ 15) for visualization only. Right-hand panels show block-averaged %ΔBOLD time courses from the subject-specific functional ROI in right M1; the gray area denotes rest and the white area denotes the active block. Participants Sub-01, Sub-02, and Sub-04 were scanned at 7T; all others at 3T. MRI images follow the radiological convention (right hemisphere shown on the left).

The functional localizer reliably engaged the motor system in all participants. Individual statistical maps had a threshold at voxel-wise FWE-corrected *p* < 0.001 with a cluster-extent threshold of k ≥ 15 voxels showed focal activation in the right primary motor cortex (M1) in every participant, with additional clusters in premotor cortex and the supplementary motor area (Figure 4). For group-level visualization, a random-effects (RFX) map is displayed at *p* < 0.005 uncorrected (k ≥ 15).

Block-averaged BOLD time courses extracted from subject-specific functional ROI in the right primary sensorimotor cortex centered on M1 (M1 ROI) exhibited the expected hemodynamic shape in seven of nine participants (Figure 4), alongside pronounced inter-individual variability in amplitude. Peak percent signal change (%ΔBOLD) ranged from approximately ~1.5% (Sub-08) to ~4.5% (Sub-02). Three participants (Sub-02, Sub-03, Sub-04) reached ≥4% peak change; two of these were scanned at 7T. These comparisons are descriptive only. Two participants deviated from the canonical pattern. In Sub-07, the time course suggested activity extending beyond the instructed tapping interval, consistent with delayed return to baseline or partial non-adherence that was not apparent online but was evident offline. In Sub-08, brief peaks at the beginning and end of the active block suggested short, punctate movements rather than sustained tapping. Despite these atypical localizer patterns, both Sub-07 and Sub-08 performed well in the subsequent neurofeedback task.

Second-level one-sample *t*-tests across subject contrast images (Active > Rest) yielded clusters of activation in the right M1. At more liberal thresholds, premotor cortex and supplementary motor area were also engaged, as were subcortical structures (putamen and pallidum) and the cerebellum, which are all consistent with the motor network (see Figure 4). Due to the modest sample size (*n* = 9) and inter-individual variability, group-level activation was less extensive spatially than suggested by several single-subject maps.

### 3.5. Individual BOLD Responses During the Neurofeedback Runs

**Figure 5:**
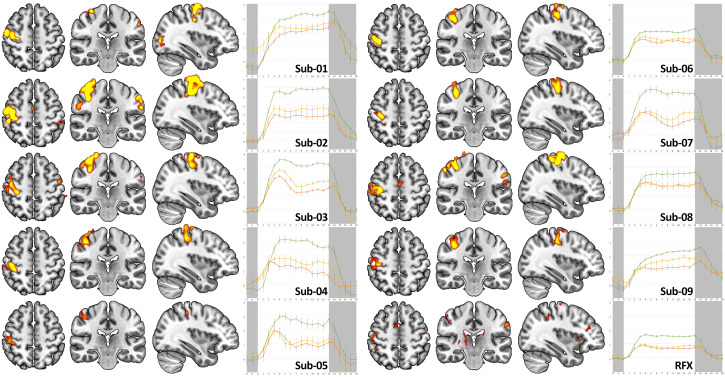
Neurofeedback runs: single-subject activation maps and block-averaged BOLD time courses. For each participant and for the RFX group analysis, statistical parametric maps (Active > Rest) are overlaid on the ICBM MNI152 template (radiological convention). Single-subject maps are thresholded at voxel-wise FWE-corrected *p* < 0.001 with k ≥ 25 (display extent). The group map is shown at *p* < 0.005 uncorrected (k ≥ 15) for visualization only. Right-hand panels plot block-averaged percent BOLD signal change from the subject-specific functional ROI in right M1 for Level 1 (red), Level 2 (yellow), and Level 3 (green); error bars denote SEM. The gray area indicates Rest and the white area the Active block. Sub-01, Sub-02, and Sub-04 were scanned at 7T; all others at 3T.

The neurofeedback runs robustly engaged the motor system across participants. Single-subject statistical maps for the contrast Active > Rest thresholded at voxel-wise FWE-corrected *p* < 0.001 with a display extent threshold of k ≥ 25 voxels showed consistent right-hemispheric clusters in the primary motor cortex (M1) in all participants, with additional involvement of premotor cortex and the supplementary motor area (Figure 5). For group-level visualization, a random-effects (RFX) map is provided at *p* < 0.005 uncorrected (k ≥ 15); this display is descriptive and not used for inference.

Block-averaged time courses from the M1 ROI extracted from subject-specific functional ROIs exhibited the expected hemodynamic rise during the active phase and a return toward baseline during the rest phase for every participant (Figure 5, right panels). As intended by the task design, response amplitudes increased with feedback level (Levels 1–3 = weak/medium/strong), with the largest step typically observed between Levels 2 and 3; in several participants, the increment from Level 1 to Level 2 was comparatively small. While cluster sizes varied across subjects, the monotonic level dependence was visually apparent in all cases. Some individuals showed a brief early negative deflection at activation onset (initial dip), whereas others reached the plateau without this feature. Notably, participants who displayed atypical localizer patterns (Sub-07, Sub-08) nevertheless showed level-dependent, time-locked responses during NFB, supporting their inclusion in the overall analyses.

When averaging hemodynamic response functions from an RFX-defined group ROI, the canonical shape was preserved but peak amplitudes were reduced and Level-1 vs. Level-2 differences were less pronounced, consistent with spatial heterogeneity across participants. These observations favor subject-specific functional ROIs for real-time fMRI neurofeedback [41].

### 3.6. Neurofeedback Performance and Learning Effects

To assess the paradigm’s feasibility, we first analyzed the overall success rate. Participants successfully modulated activity in the M1 ROI in 44.4% of all trials (216/486), confirming the task was generally achievable. A notable degree of inter-individual variability was observed, underscored by a wide performance range: the success rate in individual runs spanned from 18.5% (Participant S05, Run 1) to 74.1% (S03, Run 2).

**Figure 6:**
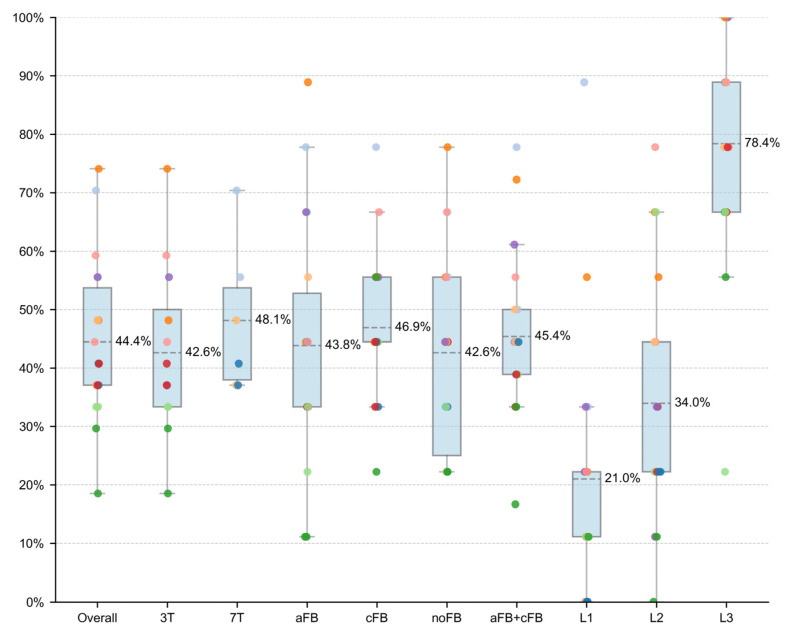
Pooled success rates across experimental categories. The figure displays neurofeedback performance for different subsets of the data. Categories include all trials (Overall), trials grouped by scanner field strength (3T, 7T), feedback modality (aFB, cFB, noFB), and target difficulty level (L1, L2, L3). Boxplots illustrate the distribution of success rates (median line, interquartile range box, whiskers at 1.5 × IQR). Each semi-transparent dot represents the success rate of a single participant (*n* = 9) for a single run (RUN1 or RUN2). Thus, each participant is represented by two dots within each category. Participant color-coding is consistent across Figure 6, Figure 7 and Figure 8. The mean success rate, aggregated across both runs, is shown as a dashed line and printed above. While performance clearly scales with target difficulty, descriptive differences between feedback modalities appear small in this sample.

To test our primary hypothesis that neurofeedback success depends on feedback type, we first analyzed performance aggregated across both experimental runs. A Generalized Linear Mixed Model (GLMM) revealed no statistically significant main effect of feedback modality on success probability (F(2, 24) = 0.514, *p* = 0.604). Given the small feasibility sample, the absence of statistical significance should be interpreted as inconclusive and does not establish equivalence between aFB, cFB, and noFB when performance is averaged over the entire session (Figure 6). This finding was corroborated by a non-parametric Friedman’s test, which also showed no significant difference between conditions (χ^2^(2) = 0.235, *p* = 0.889).

**Figure 7:**
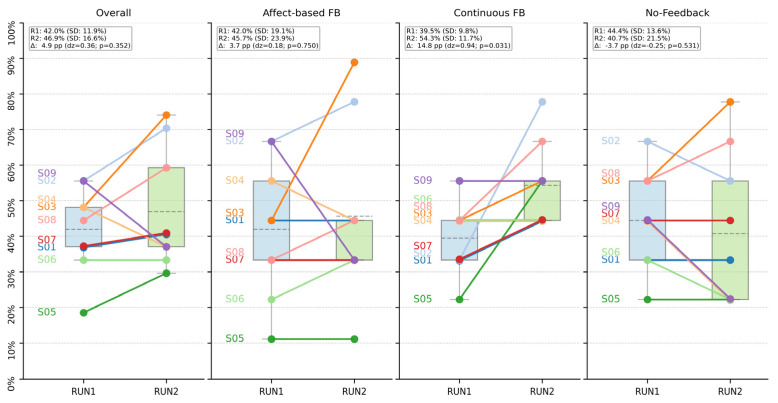
Comparison of neurofeedback success rates between two experimental runs. The figure illustrates changes in self-regulation performance for all participants (*n* = 9) between the first (RUN1) and second (RUN2) half of the session. The ‘Overall’ panel displays performance aggregated across all three feedback conditions; subsequent panels show data separated by modality: affect-based (aFB), continuous (cFB), and no-feedback (noFB). Individual participant data are shown as paired lines, and boxplots represent the group-level distribution (mean denoted by a dashed line). The inset box in each panel provides key summary statistics: it lists the mean success rate (M) and standard deviation (SD) for each run, followed by the mean paired difference between runs (Δ), the corresponding effect size (Cohen’s dz), and the *p*-value from a two-sided Wilcoxon signed-rank test.

While overall performance was similar, we next conducted our key analysis to investigate whether the feedback modalities differentially affected within-session learning. To this end, we compared success rates between the first (RUN1) and second (RUN2) halves of the experiment using paired, two-sided Wilcoxon signed-rank tests. The detailed results for all experimental subsets are visualized in Figure 7.

**Figure 8:**
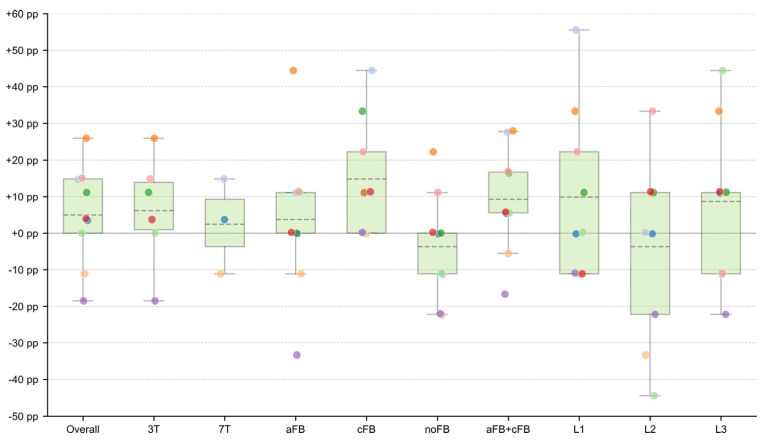
Visualization of performance change between neurofeedback runs. The figure displays the change in success rate (Δ = RUN2 − RUN1) for each experimental subset. Each colored dot represents the performance change for a single participant; colors are consistent with the participant identities shown in Figure 7. Positive values indicate an improvement in performance from the first to the second run. The background boxplots summarize the distribution of these paired differences for each category (median line, interquartile range box, whiskers at 1.5 × IQR). The mean change is indicated by a dashed line. The distribution for the continuous feedback (cFB) condition is visibly shifted upwards compared to the affect-based (aFB) and no-feedback (noFB) conditions, illustrating the modality-specific learning effect.

Our central finding is a statistically significant and large learning effect that was exclusively present for the continuous feedback (cFB) condition. As shown in Figure 7, performance for cFB improved by a mean of +14.8 percentage points, rising from 39.5% (SD = 9.8%) in RUN1 to 54.3% (SD = 11.7%) in RUN2. This increase was statistically significant (*p* = 0.031) and represented a large effect size (Cohen’s d_z = 0.94) [42]. Figure 8 provides a direct visualization of these performance changes (Δ = RUN2 − RUN1) across all experimental subsets, highlighting the pronounced positive shift specifically for the cFB condition.

In stark contrast, no evidence of learning was found for the other feedback modalities. For the affect-based feedback (aFB) condition, performance remained stable, with a non-significant change of +3.7 percentage points (*p* = 0.75, d_z = 0.18; Figure 7). Similarly, the no-feedback (noFB) transfer condition, designed to assess regulation without external cues, showed a slight, non-significant decrease in performance (Δ = −3.7 pp, *p* = 0.531, d_z = −0.25; Figure 7), indicating that participants were able to maintain their previously acquired skill but did not improve further. When aggregating across all conditions (Figure 7), the small mean improvement of +4.9 percentage points was not statistically significant (*p* = 0.352, d_z = 0.36), underscoring that the significant learning effect was specific to the cFB modality.

The continuous metrics yielded the same qualitative pattern as the binary success rate. For continuous thermometer feedback (cFB), all three measures improved from RUN1 to RUN2 (median distance-to-range: 4.63 ± 4.16 to 3.81 ± 6.80, *p* = 0.641; distance-to-range SD: 9.70 ± 3.24 to 9.10 ± 3.34, *p* = 0.203; occupancy: 0.443 ± 0.060 to 0.476 ± 0.115, *p* = 0.301). In contrast, affect-based face feedback (aFB) and the no-feedback condition (noFB) showed mixed and non-convergent changes across metrics (all *p* ≥ 0.25), indicating no clear continuous-learning trend that would have been masked by the binary threshold.

Exploratory analyses stratified by scanner field strength revealed no significant learning effects for either the 3T participants (*p* = 0.438) or the 7T participants (*p* = 0.750), though these comparisons are limited by the small and unbalanced sample sizes. Furthermore, when analyzed by target difficulty, no significant learning was detected for weak (Level 1), medium (Level 2), or strong (Level 3) activation targets (*p* > 0.35 for all). The particularly high baseline performance for Level 3 (RUN1 M = 74.1%) likely produced a ceiling effect, limiting the potential to observe further improvement.

### 3.7. Offline Group Analysis of fMRI Data

Using the preprocessed whole-brain data and first-level contrast images described in Section 2.4, we conducted an exploratory offline random-effects (RFX) group analysis to characterize activation patterns associated with the different neurofeedback conditions. Group maps are displayed at voxel-wise *p* < 0.001 (uncorrected) with a cluster-extent threshold of k ≥ 15 voxels. Given the modest sample size (*n* = 9) and the liberal threshold, these whole-brain results are reported descriptively. Detailed tables of activated areas including peak MNI coordinates, cluster sizes, and their anatomical labeling are provided in the Supplementary Material.

**Figure 9:**
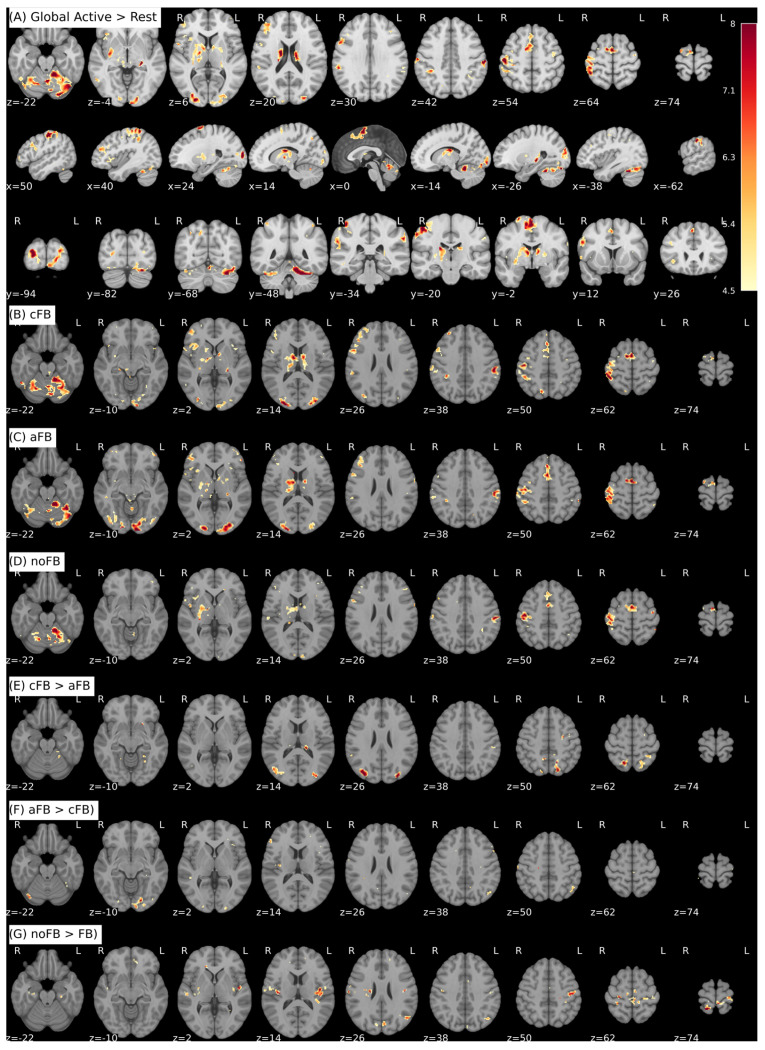
Offline fMRI group analysis (RFX, N = 9). All maps are shown for exploratory/descriptive visualization only (small sample size; uncorrected thresholding); no corrected whole-brain inference is claimed. All maps are thresholded at voxel-wise *p* < 0.001 (uncorrected) with a cluster-extent threshold of k ≥ 15 voxels. Statistical parametric maps are overlaid on the ICBM MNI152 template (radiological convention). (**A**) The main effect of regulation (global activity > rest) as observed in three orthogonal planes across all trials and conditions. It shows the expected activation pattern primarily in the motor system (primary sensorimotor cortex areas [SMC], supplementary motor area [SMA], basal ganglia, and cerebellum) as well as in the visual cortex. (**B**–**D**) Main effect for cFB (**B**), aFB (**C**), and noFB (**D**) conditions vs. baseline. (**E**) Differential contrast cFB > aFB, clusters were primarily located in dorsal occipital and parietal regions, including bilateral superior occipital gyrus and precuneus. (**F**) Differential contrast aFB > cFB, showing clusters in ventral temporal and frontal regions, including the left fusiform gyrus, left anterior hippocampus, and orbitofrontal/medial frontal regions. (**G**) Contrast noFB > FB, showing increased activation in bilateral sensorimotor cortex and SMA, with notably additional recruitment of the ipsilateral (left) hemisphere, as well as in higher-order association areas including the angular gyrus and middle temporal gyrus.

For a first validation of the experimental paradigm at the whole-brain level, we examined the main activation pattern across all regulation blocks collapsed over feedback modality (Figure 9A). As expected, the contrast Global Active > Rest yielded robust activation in a core motor network consisting of the primary sensorimotor cortices (SMC, contralateral > ipsilateral), supplementary motor area (SMA), basal ganglia, and cerebellum, with the strongest cluster centered in contralateral (right) SMC, corresponding to the predefined neurofeedback target region.

We then inspected condition-specific activation patterns. Both visual feedback conditions (cFB and aFB; Figure 9B,C) showed similar recruitment of the motor system but additionally exhibited widespread clusters in the occipital cortex and higher-order visual areas. In the no-feedback transfer condition (noFB; Figure 9D), activation of the motor system during regulation was comparable, while no pronounced clusters were observed in early or higher-order visual regions relative to baseline at the chosen threshold.

To assess the neural substrates underlying the different feedback types we contrasted the two visual modalities. The contrast cFB > aFB revealed stronger activation within dorsal occipital and parietal regions, with prominent bilateral clusters in the superior occipital gyri and precuneus (Figure 9E). In the opposite direction, the contrast aFB > cFB yielded smaller and more spatially restricted clusters (Figure 9F), including peaks in the left fusiform gyrus, left anterior hippocampus, and additional clusters in orbitofrontal and inferior medial frontal cortices.

Finally, to identify the neural correlates of regulating motor output based purely on internal processes, we compared the no-feedback condition with the combined visual feedback conditions (noFB > FB). This contrast revealed clusters in the bilateral SMC and SMA, as well as in higher-order association areas, including the left angular gyrus and the temporal lobes (Figure 9G). Interestingly, additional neuronal ensembles were recruited in the ipsilateral (left) SMC (differential activation SMC L > SMC R).

## 4. Discussion

In this feasibility study, we examined how different visual feedback designs affect learning of volitional self-regulation of a right primary sensorimotor cortex ROI centered on the primary motor hand area (M1 ROI) in real-time fMRI. Participants were able to successfully modulate activity in this M1 ROI across all three experimental conditions. It should be noted that we did not differentiate between the primary motor and primary sensory areas, as we used both together as a target area. We assumed that sensory feedback would increase with more vigorous finger movements, meaning that S1 would also show increased activation.

Surprisingly, a robust within-session learning effect emerged only for the continuous, signal-proportional thermometer feedback (cFB), whereas neither the affect-based face feedback (aFB) nor the no-feedback transfer condition (noFB) showed comparable improvement (cf. Figure 7 and Figure 8). These findings indicate that, within a single-session training protocol, the design of the feedback display is a critical factor shaping the acquisition of a neurofeedback skill [9]. Notably, secondary analyses using continuous, task-congruent distance-to-range and occupancy metrics confirmed the binary findings and did not reveal masked trend-level learning in the aFB or noFB conditions.

When aggregating performance across both runs, we did not observe a significant main effect of feedback modality in a generalized linear mixed-effects model, and this null result was robust across different model specifications and corroborated by a non-parametric Friedman test (cf. Figure 6 for the descriptive distribution across modalities). In the context of a small-sample feasibility study, this absence of an overall performance difference is difficult to interpret: it may reflect genuinely similar average performance across modalities, but it is equally compatible with a lack of statistical power to detect small-to-medium effects. Accordingly, we do not interpret the non-significant main effect as evidence of equivalence between modalities; rather, larger confirmatory studies are needed to quantify potential small-to-medium differences in overall success. Importantly, however, the within-session learning effect [1] for cFB was large and consistent across participants, while neither aFB nor noFB showed analogous improvement. Thus, while we refrain from strong conclusions about absolute differences in overall performance, our data clearly indicate that a continuous, signal-proportional display particularly facilitates rapid learning within a short training protocol.

A straightforward explanation for the superior learning with cFB lies in the informational content and granularity of the feedback signal. The thermometer provides a direct, monotonic mapping between the underlying BOLD signal in the SMC ROI and the visual display, allowing participants to continuously fine-tune their strategies and immediately observe small improvements or deteriorations, a process central to skill acquisition [13]. By contrast, the aFB display compresses the signal into a small number of categorical levels. This lack of granularity complicates fine-tuning and introduces interpretive ambiguity. The additional need to decode a symbolic social cue—for example, to interpret the valence and congruence of a smiling face in relation to one’s motor strategy—likely imposes extra cognitive load [19]. This interpretation is consistent with our offline group-level fMRI analysis, where aFB, compared with cFB, recruited regions outside the core visuo-motor loop, including fusiform gyrus for face processing [43] and hippocampus for associative memory [44], whereas cFB preferentially engaged dorsal occipital and parietal regions associated with visuospatial processing and sensorimotor integration [45].

Beyond our paradigm, there is converging evidence that more complex, socially meaningful feedback signals are not necessarily advantageous for neurofeedback learning. Recent work by Watve et al. [18] utilized highly sophisticated, dynamic face morphing for amygdala regulation and found significant learning effects only in a specific subgroup (fear reduction), while failing to observe robust learning in positive affect conditions. This pattern aligns with our finding that social-emotional feedback adds an additional layer of interpretative complexity—such as decoding valence or congruence—that may hinder rapid skill acquisition compared to the distinct clarity of a signal-proportional thermometer. Even when face feedback is continuous and biologically plausible for the target region, as in Watve et al.’s amygdala study, it does not guarantee superior learning. In our motor cortex paradigm, where the social cue is arbitrary with respect to the regulated SMC ROI, this disadvantage appears even more pronounced.

A core goal of neurofeedback is to help individuals develop an internal model [1] of self-regulation that can be used without external feedback. In our noFB condition, participants received explicit success feedback only at the end of each block, yet they were still able to volitionally modulate activity in the SMC ROI during the interaction phase by relying on interoceptive and proprioceptive cues alone. This suggests that participants began to form an internal control model that did not strictly depend on the visual display. Importantly, the noFB condition was not visually matched to the feedback conditions (static fixation vs. dynamically updating displays) and therefore differed not only in feedback contingency but also in visual stimulation and attentional demands. Consequently, comparisons involving noFB should be interpreted cautiously, as differences cannot be attributed uniquely to feedback availability. However, we did not observe a clear learning effect between runs in the noFB condition, indicating that a single session with intermittent feedback is not sufficient to consolidate this skill. This interpretation is in line with larger multi-study work showing that performance in pre-training no-feedback runs does not reliably predict subsequent learning with feedback and highlighting that the presence of an online feedback signal fundamentally changes both the task and the learning process [20]. Moreover, machine-learning analyses across 28 real-time fMRI neurofeedback experiments suggest that simply including a pre-training no-feedback run—regardless of individual performance in that run—is one of the few paradigm-level design features associated with higher overall neurofeedback performance [21]. Multi-session studies will be needed to track the consolidation of such internal models, assess long-term retention, and test generalization to tasks without overt movement or without any visual feedback.

In order to optimize the feedback strategies and further elucidate the neural mechanisms underlying these behavioral differences, the offline random-effects analysis contrasted the different activation patterns. As expected, the main effect of regulation (Global Active > Rest) revealed robust, widespread activation patterns encompassing core components of the sensorimotor system, including the sensorimotor cortex (SMC), supplementary motor area (SMA), basal ganglia and cerebellum, across all conditions. This also serves as a quality control measure to verify that the experimental setup is functioning as intended.

The differential contrasts provide more insights into how feedback design shapes neural processing. Contrast cFB > aFB (Figure 9E) showed more extensive clusters/stronger cluster expression at the chosen threshold in the dorsal stream of the visual system, particularly in the superior occipital gyrus and precuneus. These regions are well known for their involvement in visuo-spatial processing and handling quantitative information. This is consistent with the task demands of the continuous feedback condition, in which participants must continually monitor the height and position of the thermometer bar in order to guide their motor performance.

Conversely, the aFB > cFB contrast produced only a few clusters of activation (see Figure 9F). Notably, however, we observed activation in the left fusiform gyrus, a region classically associated with face processing, and in the left anterior hippocampus. The latter may reflect the recruitment of memory and associative processes required to interpret the social meaning of the smiling face, or to map the categorical facial expression onto the internal motor state. Furthermore, we observed activation in frontal regions, including parts of the inferior/middle orbitofrontal cortex (OFC) and the inferior frontal medial cortex. The OFC is functionally connected to limbic structures and plays a central role in emotion regulation and social behavior. Although the activation was less extensive than that observed in the dorsal stream for cFB, this pattern suggests that affect-based feedback indeed engages additional cognitive and emotional processing loops. It is plausible that stimuli that are more emotionally salient than our standardized, gender-neutral faces might elicit stronger activation in these regions in future studies.

At the core of neurofeedback is the question of whether relying on visual feedback changes the neural underpinnings of the motor task itself. Differential contrast analysis of the no-feedback transfer condition against both visual feedback conditions combined (noFB > FB) suggested relatively stronger involvement/more pronounced clusters primarily within the sensorimotor system itself (see Figure 9G) and in the left angular and temporal gyri. To avoid any misunderstandings, it should be noted that the basal SMC activation is still stronger contralaterally than ipsilaterally, and comparable for both conditions, but the additional relative activation is stronger ipsilaterally than contralaterally. The angular and temporal regions implicated in proprioception and multisensory integration are evident here. The more pronounced upregulation of the ipsilateral SMC than the contralateral SMC suggests that, in the absence of external visual cues, participants relied more heavily on internal sensorimotor and interoceptive representations to guide their movements. This increased ipsilateral recruitment may indicate a compensatory mechanism or higher demand on the motor network to maintain the ‘internal model’ of the task without the ‘visual crutch’ of the feedback signal. This finding is consistent with evidence from stroke rehabilitation suggesting that reliance on internal representations can modulate the extent and lateralization of motor network activation.

Finally, we would like to point to the fact that a stable and low-latency data-processing pipeline is a critical prerequisite for valid and effective real-time fMRI neurofeedback [8,9]. In our setup, the total end-to-end latency, with a mean of approximately 794 ms, remained well within the 2 s TR, ensuring that feedback was, on average, presented without gross delay. Nevertheless, our detailed latency analysis revealed stochastic outliers, particularly during 7T sessions, where transfer times occasionally exceeded the TR. Such unpredictable data delays are not unique to our system and have been described in other implementations as an initial “chaotic phase” followed by a more stable but still imperfect phase of image reconstruction and export [46]. High computational load for multiband reconstruction (particularly for the first image of a multiband sequence), combined with limitations in scanner data-export infrastructure and network protocols (e.g., legacy SMB-based file sharing), are likely contributors [46]. Direct scanner-to-analysis connections as proposed by Lührs et al. [47] offer one promising strategy to substantially reduce transfer times and avoid such bottlenecks. In our study, trials affected by excessive transfer delays were excluded from the group-level analyses, which had only minimal impact on the main results. However, for future applications—especially interactive paradigms or hyperscanning scenarios [48,49]—real-time monitoring of transfer times and automated exclusion or replacement of delayed trials will be necessary to maintain data quality and ensure interpretable feedback. Multi-site real-time fMRI frameworks require not only robust data transfer but also cross-site signal calibration to account for hardware heterogeneity [49].

Our study has several methodological limitations that constrain the generalizability of the findings. First, the sample size was modest (*n* = 9), and participants were not balanced across scanner field strengths. Moreover, allocation to 3T versus 7T was non-random (scanner availability) and may confound scanner-related effects with subject-group differences; thus, field-strength comparisons should be interpreted as exploratory. Consequently, the study was not powered to reliably detect small-to-medium effects, particularly in between-group comparisons such as 3T versus 7T. Second, our primary performance metric was a binary success indicator based on a simple a priori threshold (≥50% of regulation TRs within the target level). While this operationalization is intuitive and suitable for feasibility work, it may obscure more subtle differences that could emerge when using continuous measures such as mean deviation from target levels, the slope of learning curves, or model-based performance indices. To mitigate this limitation, we additionally performed a sensitivity analysis using continuous, task-congruent occupancy and distance-to-range metrics, which reproduced the qualitative pattern observed with the binary outcome. Importantly, this limitation is shared with much of the current real-time fMRI neurofeedback literature: there is still no consensus on a gold-standard definition of neurofeedback learning, and recent meta- and mega-analyses therefore had to rely on multiple, partly non-equivalent success measures when aggregating results across studies [14,20,21]. A complementary systematic review focusing specifically on clinical rt-fMRI-NFB trials similarly concluded that most existing studies are small, heterogeneous and often underpowered, with limited consistency in reporting core clinical and regulation outcomes [22]. Third, the feedback signal was normalized to each participant’s localizer maximum (100%) and values above 100% were clipped for the display. While this scaling was intentionally used to standardize task scaling across individuals and across 3T and 7T sessions, it may introduce between-subject scaling differences because identical relative targets can correspond to different absolute %ΔBOLD amplitudes. Moreover, display saturation at high activation levels could compress improvements above the localizer maximum, potentially attenuating apparent learning effects at the upper end of the scale. Future work should systematically compare different performance metrics within the same datasets to clarify which measures are most sensitive and most predictive of clinically or behaviorally meaningful change.

We sought to align the design and reporting of this feasibility study with recent consensus recommendations for neurofeedback (CRED-nf) [25] and for MRI data analysis and sharing (COBIDAS-MRI) [39]. Nevertheless, several elements emphasized in these guidelines could not be fully implemented here and should be addressed in future confirmatory trials. For example, we did not include long-term follow-up assessments, transfer tasks without overt movement, or systematic manipulation of learning strategies. Moreover, while we focused on a relatively robust and well-understood target region (the M1 ROI defined by overt finger tapping), our results may not directly generalize to paradigms targeting higher-order cognitive or affective regions. The mapping between mental strategies, neural signals, and feedback displays is likely more complex and less deterministic in such cases, posing a significant challenge for the field [14].

Despite these limitations, our findings allow several practical recommendations. For studies aiming to teach neural self-regulation quickly within a limited number of runs, a clear, continuous feedback display appears most effective in our data. If more complex or socially meaningful feedback is employed, its additional interpretive demands should be carefully considered, as they may slow down early learning despite potential long-term motivational benefits. Our results further highlight the importance of monitoring and optimizing end-to-end latency and its variability, particularly when moving to ultra-high field systems or more demanding real-time applications. Finally, future work should extend the present feasibility results by (i) increasing sample sizes and statistical power; (ii) systematically comparing different feedback designs across multiple sessions; and (iii) assessing the extent to which learned regulation of the used target ROI transfers to tasks without overt feedback and to clinically relevant contexts such as motor rehabilitation. Consequently, these steps are imperative in translating design choices in real-time fMRI neurofeedback from technical possibilities into evidence-based recommendations.

## 5. Conclusions

Based on our findings, we can derive several practical recommendations for future neurofeedback studies:

1. Prioritize clarity for rapid learning: For studies aiming to teach neural self-regulation quickly (e.g., in a single session), a clear, continuous, and signal-proportional feedback display (like a thermometer) appears superior to categorical or social cues.
2. Simplify engaging feedback: If motivation-enhancing feedback (like faces) is required—for instance, in pediatric populations or multi-session clinical trials—its design should be simplified. Reducing the number of target levels or using abstract icons might mitigate the cognitive load associated with decoding complex social signals.
3. Model Systems for Technical Validation: The primary sensorimotor cortex proved to be a robust model system for validating the technical setup across field strengths. Because sensorimotor activation is strong and reliable, it allows researchers to disentangle technical issues (latency, signal quality) from the psychological complexity of learning, which is harder to achieve in cognitive or emotional networks.

In summary, this study demonstrates the technical feasibility of multi-field strength neurofeedback and underscores that “fancier” feedback is not always better. For rapid skill acquisition, the brain appears to benefit most from the precise, unadulterated information provided by a simple gauge.

## Data Availability

The Turbo-BrainVoyager plug-ins used in this study are publicly available via DOI: 10.5281/zenodo.10356620 (INIT) and 10.5281/zenodo.10370720 (NFB). All other data supporting the findings of this study (including raw MRI data, participant-level data/metadata, and any additional materials) are available from the corresponding author upon reasonable request due to ethical and data protection restrictions.

## Supplementary Materials

The following supporting information can be downloaded at: https://www.mdpi.com/article/10.3390/brainsci16020166/s1, Figure S1: Computational processing times; Figure S2: Total system latency (end-to-end); Figure S3: Detailed visualization of self-regulation performance; Table S1.1: Global Active > Rest; Table S1.2: cFB > rest; Table S1.3: aFB > rest; Table S1.4: noFB > rest; Table S1.5: cFB > aFB; Table S1.6: aFB > cFB; Table S1.7: noFB > FB.

## Abbreviations

The following abbreviations are used in this manuscript:

aFB	Affect-based categorical feedback
AICC	Corrected Akaike Information Criterion
BOLD	Blood-oxygenation-level-dependent
cFB	Continuous signal-proportional feedback
CI	Confidence interval
COBIDAS-MRI	Best Practices in Data Analysis and Sharing in Neuroimaging using MRI
CRED-nf	Consensus on the Reporting and Experimental Design of neurofeedback
EEG	Electroencephalography
EPI	Echo-planar imaging
fMRI	Functional magnetic resonance imaging
FWE	Family-wise error
FWHM	Full width at half maximum
GLM	General linear model
GLMM	Generalized Linear Mixed Model
IQR	Interquartile range
M1	Primary motor cortex
MNI	Montreal Neurological Institute
MRI	Magnetic resonance imaging
NFB	Neurofeedback
noFB	No-feedback transfer condition
OFC	Orbitofrontal cortex
RFX	Random-effects
ROI	Region of interest
rt-fMRI	Real-time functional magnetic resonance imaging
SD	Standard deviation
SEM	Standard error of the mean
SMA	Supplementary motor area
SMC	Primary sensorimotor cortex
TBV	Turbo-BrainVoyager
TE	Echo time
TR	Repetition time
