# Supplementary material for: The Impact of Visual Feedback Design on Self-Regulation Performance and Learning in a Single-Session rt-fMRI Neurofeedback Study at 3T and 7T
Source: Brain Sci. 2026 Jan 30;16(2):166. doi: 10.3390/brainsci16020166 (PMC12938616; doi:10.3390/brainsci16020166)
Supplement: Supplementary file 1 [file brainsci-16-00166-s001.zip › brainsci-4105064-supplementary.pdf]

Supplementary Material

The impact of visual feedback design on self-regulation performance and learning in a single-session rt-fMRI neurofeedback study at 3T and 7T

Sebastian Baecke, Ralf Lützkendorf, Johannes Bernarding

Data transfer and processing times

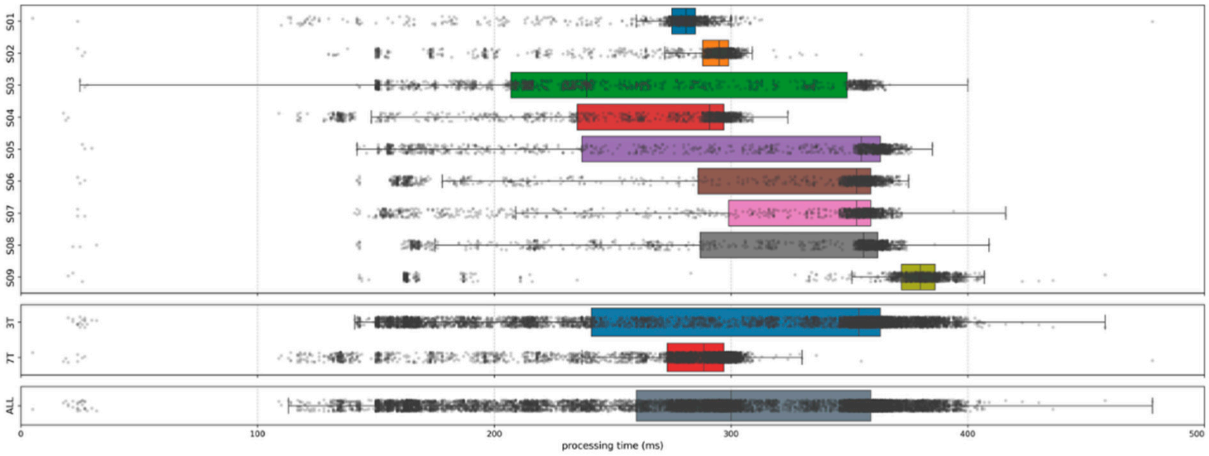

Figure S1: *Computational processing times. Boxplots illustrating the processing time required by the neurofeedback workstation (real-time preprocessing, GLM calculation, and feedback rendering) in milliseconds. Top: Individual processing times for all subjects (S01–S09). Middle: Data aggregated by magnetic field strength. Note that processing time is largely independent of field strength. Bottom: Aggregated data for all volumes. The vertical lines (whiskers) indicate the minimum and maximum values excluding outliers. The mean processing time was 296.8 ms (99% CI: 295.1–298.5 ms).*

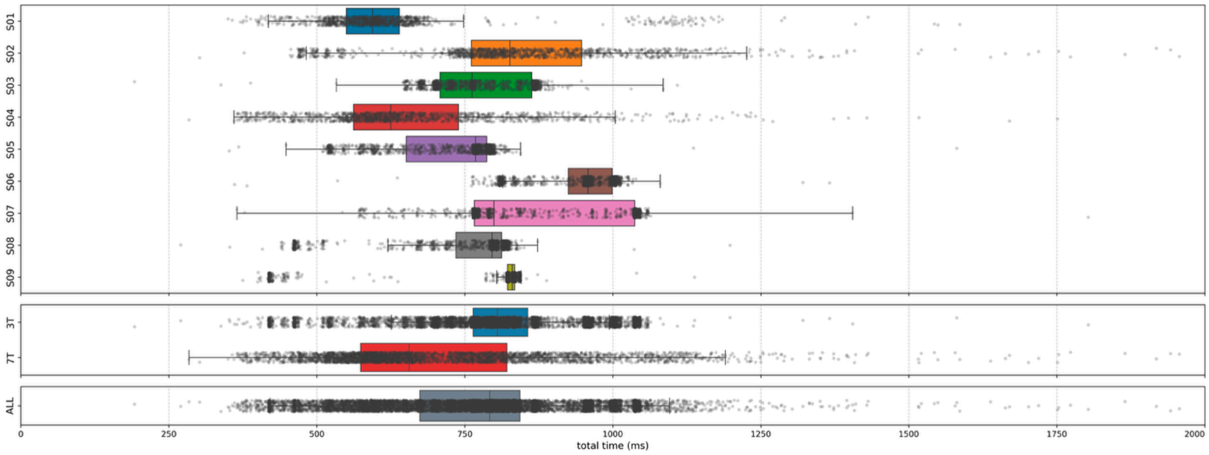

Figure S2: *Total system latency (End-to-End). Boxplots showing the sum of transfer/reconstruction time (see Figure 3 in main text) and computational processing time (Figure S1). This represents the total delay from acquisition of the last slice to the update of the visual display. Top: Individual subject latencies. Note the increased variance and outliers for 7T subjects (S01, S02, S04). Middle: Aggregated by field strength. Bottom: Global aggregation. Despite outliers at 7T, the median total latency remained well below the TR of 2000 ms.*

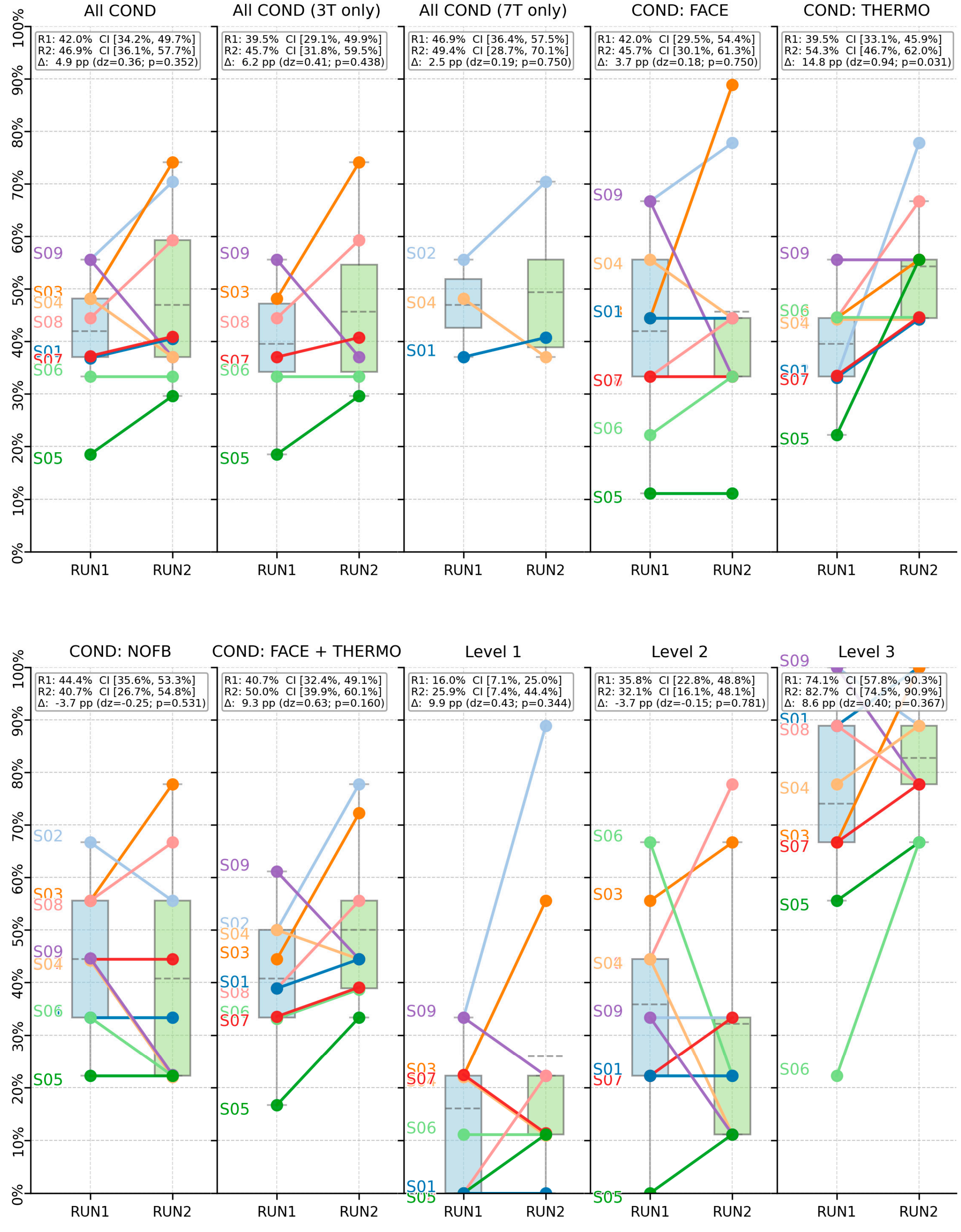

Figure S3: *Detailed visualization of self-regulation performance. Individual success rates (dots) and group-level trends (boxplots) separated by experimental condition and target difficulty. Top Row: Performance split by feedback modality (aFB, cFB, noFB) and comparison of run 1 vs. run 2. Bottom Row: Performance split by target activation level (Level 1 = weak, Level 2 = medium, Level 3 = strong).*

Offline Group analysis

(cf. Fig. 9, main text)

Table S1.1 : Global Active > Rest

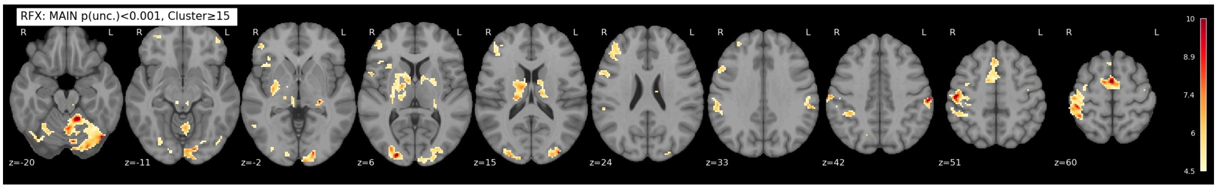

X	Y	Z	Region	Size	PeakT	MeanT	StdT
**-26**	-26	-4	Hippocampus_L (15%), Thal_MGN_L (11%)	88	21.33	5.88	2.07
**52**	-20	54	Parietal_Sup_R (43%), Precentral_R (17%), Angular_R (17%), SupraMarginal_R (13%), Parietal_Inf_R (5%)	1,398	19.10	5.63	1.22
**-14**	-48	-22	Cerebellum_7b_L (24%), Occipital_Mid_L (19%), Cerebellum_6_L (15%), Cerebellum_Crus2_L (13%), Calcarine_L (7%), Fusiform_L (7%)	2,419	18.69	5.94	1.39
**0**	-2	62	Supp_Motor_Area_R (55%), Rolandic_Oper_L (30%), Frontal_Sup_2_R (10%)	861	15.32	5.81	1.36
**24**	-94	6	Occipital_Inf_R (46%), Occipital_Mid_R (18%), Lingual_R (11%), Cuneus_R (10%), Occipital_Sup_R (8%)	452	13.86	5.93	1.46
**-62**	-24	42	SupraMarginal_L (67%), Parietal_Inf_L (20%)	196	13.12	6.23	1.61
**20**	-22	18	Pallidum_R (16%), Putamen_R (13%), Thal_IL_R (10%), Thal_Re_R (9%), Heschl_R (7%)	1,027	11.71	5.42	0.92
**-12**	-12	22	Caudate_L (19%), Thal_VPL_L (19%), Putamen_L (16%), Thal_VL_L (6%)	341	9.79	5.35	0.85
**44**	-50	-28	Cerebellum_8_R (69%), Postcentral_R (14%), Cerebellum_3_R (10%)	604	9.03	5.48	0.83
**54**	12	30	Frontal_Inf_Oper_R (78%), Precentral_R (22%)	193	7.54	5.25	0.68
**6**	-78	-26	Vermis_10 (73%), Cerebellum_3_R (20%)	44	6.90	5.24	0.54
**40**	44	22	Frontal_Mid_2_R (72%), Frontal_Inf_Tri_R (27%)	408	6.89	5.21	0.53
**16**	-70	44	Paracentral_Lobule_R (95%), Parietal_Inf_R (5%)	20	6.83	5.44	0.58
**54**	8	6	Frontal_Inf_Oper_R (52%), Rolandic_Oper_R (48%)	31	6.60	5.17	0.53
**58**	-58	-6	Cerebellum_Crus2_R (71%), Temporal_Inf_R (29%)	35	6.26	5.18	0.52
**40**	28	-4	Insula_R (72%), Frontal_Inf_Tri_R (15%), Frontal_Inf_Orb_2_R (5%)	150	6.20	4.91	0.36
**30**	46	34	Frontal_Mid_2_R (95%), Frontal_Sup_2_R (5%)	20	5.93	5.00	0.40
**-40**	-12	64	Background (100%)	34	5.83	4.86	0.33
**-44**	50	-14	Frontal_Sup_2_L (56%), OFCpost_L (30%)	27	5.60	4.95	0.32
**30**	54	-14	Frontal_Mid_2_R (46%), OFCant_R (32%), Frontal_Sup_2_R (22%)	37	5.57	4.93	0.31
**38**	-4	52	Precentral_R (51%), Frontal_Sup_2_R (29%), Frontal_Mid_2_R (20%)	35	5.46	4.80	0.24

Table S1.2 : *cFB > rest*

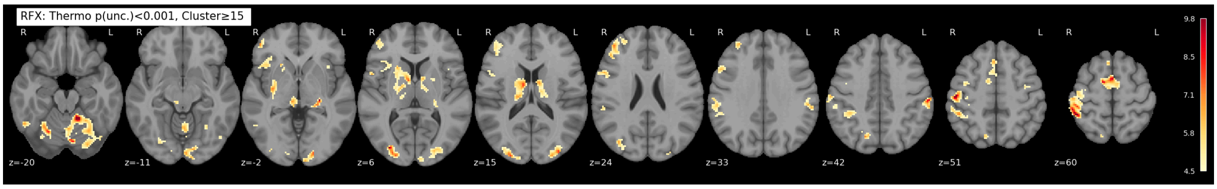

X	Y	Z	Region	Size	PeakT	MeanT	StdT
**50**	-20	54	Parietal_Sup_R (34%), Angular_R (26%), SupraMarginal_R (15%), Precentral_R (13%), Parietal_Inf_R (5%)	1,270	17.07	5.56	1.30
**-14**	-46	-22	Cerebellum_7b_L (40%), Cerebellum_6_L (24%), Cerebellum_Crus2_L (15%)	1,461	15.39	5.92	1.38
**-26**	-24	-4	Hippocampus_L (23%), Thal_MGN_L (17%), Thal_PuM_L (7%)	102	13.38	5.97	1.41
**10**	-2	62	Supp_Motor_Area_R (56%), Rolandic_Oper_L (26%), Frontal_Sup_2_R (14%)	778	13.12	5.63	1.20
**32**	-84	6	Occipital_Inf_R (70%), Occipital_Mid_R (15%), Lingual_R (8%)	452	10.90	5.76	1.20
**-56**	-28	38	SupraMarginal_L (66%), Parietal_Inf_L (21%)	226	9.92	5.81	1.22
**16**	-8	20	Pallidum_R (14%), Putamen_R (13%), Thal_VPL_L (9%), Thal_IL_R (6%), Caudate_L (6%)	1,336	9.82	5.39	0.85
**22**	-64	-22	Cerebellum_8_R (83%), Postcentral_R (11%)	463	9.22	5.56	0.90
**-18**	-90	-4	Occipital_Mid_L (61%), Calcarine_L (21%), Lingual_L (8%)	639	8.92	5.53	0.80
**42**	44	22	Frontal_Mid_2_R (78%), Frontal_Inf_Tri_R (20%)	415	8.58	5.33	0.71
**32**	44	36	Frontal_Mid_2_R (89%), Frontal_Sup_2_R (11%)	47	8.25	5.45	0.83
**18**	-68	40	Paracentral_Lobule_R (69%), Parietal_Inf_R (20%), Occipital_Mid_R (10%)	135	8.11	5.36	0.72

**42**	20	-6	Insula_R (77%), Frontal_Inf_Tri_R (9%)	225	8.00	5.20	0.55
**52**	-52	-22	Cerebellum_Crus2_R (97%)	34	7.32	5.49	0.77
**-16**	-6	72	Precentral_L (72%), Background (17%), Rolandic_Oper_L (11%)	18	7.19	5.27	0.67
**56**	10	28	Frontal_Inf_Oper_R (68%), Precentral_R (21%), Rolandic_Oper_R (6%)	297	7.06	5.20	0.57
**22**	-28	-10	ParaHippocampal_R (80%), Occipital_Sup_R (20%)	20	6.48	5.32	0.59
**34**	54	26	Frontal_Mid_2_R (60%), Frontal_Sup_2_R (40%)	47	5.79	4.97	0.36
**38**	-4	52	Frontal_Mid_2_R (50%), Precentral_R (47%)	32	5.70	4.84	0.32
**54**	-60	-6	Cerebellum_Crus2_R (71%), Temporal_Inf_R (29%)	31	5.68	4.89	0.32
**-22**	-76	-8	Fusiform_L (80%), Lingual_L (20%)	15	5.61	4.90	0.33
**-34**	14	12	OFClat_L (85%), Frontal_Inf_Oper_L (15%)	26	5.60	4.80	0.25
**-36**	-8	52	Background (100%)	19	5.45	4.82	0.24
**14**	-92	-4	Occipital_Sup_R (64%), Cuneus_R (36%)	22	5.24	4.81	0.20
**-22**	0	10	Putamen_L (100%)	28	5.06	4.72	0.17
**-36**	18	0	OFClat_L (96%)	27	4.91	4.63	0.11

Table S1.3 : ***aFB* > *rest***

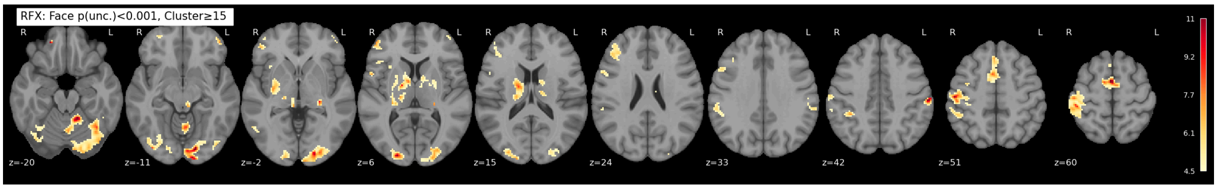

X	Y	Z	Region	Size	PeakT	MeanT	StdT
**-38**	-54	-24	Occipital_Mid_L (21%), Cerebellum_7b_L (15%), Cerebellum_6_L (13%), Fusiform_L (12%), Cerebellum_Crus2_L (11%), Calcarine_L (7%), Lingual_L (7%), Occipital_Inf_L (6%)	2,384	20.67	6.12	1.66
**52**	-20	54	Parietal_Sup_R (50%), Precentral_R (21%), SupraMarginal_R (12%), Angular_R (7%), Parietal_Inf_R (5%)	1,124	20.19	5.69	1.29
**0**	-2	62	Supp_Motor_Area_R (61%), Rolandic_Oper_L (33%)	617	16.56	5.84	1.46
**-62**	-24	44	SupraMarginal_L (68%), Parietal_Inf_L (11%)	167	15.27	6.07	1.67
**-24**	-30	4	Hippocampus_L (13%), Thal_MGN_L (12%)	60	13.08	6.23	1.61
**22**	-94	4	Cerebellum_8_R (23%), Occipital_Inf_R (13%), Postcentral_R (13%), Cuneus_R (11%), Fusiform_R (10%), Occipital_Mid_R (7%), Occipital_Sup_R (6%)	1,098	10.87	5.42	0.92
**8**	-4	12	Pallidum_R (16%), Thal_IL_R (13%), Putamen_R (12%), Thal_Re_R (10%), Heschl_R (7%), Thal_VPL_R (5<%)	928	10.58	5.58	1.01
**20**	48	-20	OFCant_R (67%), Frontal_Mid_2_R (19%), OFCmed_R (12%)	42	10.44	5.48	1.05
**-16**	-18	20	Caudate_L (22%), Putamen_L (17%), Thal_VPL_L (12%), Thal_VL_L (8%), Pallidum_L (7%)	312	9.76	5.31	0.82
**8**	-32	-6	Thal_LGN_R (5%)	37	8.58	5.24	0.89
**6**	-80	-26	Cerebellum_3_R (41%), Vermis_10 (41%), Cerebellum_4_5_R (14%)	22	8.45	5.74	1.32
**50**	46	4	Frontal_Mid_2_R (68%), Frontal_Inf_Tri_R (28%)	437	7.72	5.28	0.61
**-46**	48	-12	Frontal_Sup_2_L (63%), OFCpost_L (20%)	35	7.39	5.27	0.73
**56**	12	30	Frontal_Inf_Oper_R (84%), Precentral_R (16%)	139	7.27	5.21	0.57
**54**	8	6	Rolandic_Oper_R (52%), Frontal_Inf_Oper_R (48%)	27	7.15	5.31	0.61
**58**	-36	32	Angular_R (91%), Temporal_Mid_R (9%)	138	7.12	5.04	0.53
**22**	6	72	Frontal_Sup_2_R (83%), Supp_Motor_Area_R (7%)	29	6.51	5.18	0.58
**-44**	-42	-16	Cerebellum_Crus1_L (100%)	16	6.08	5.17	0.46
**48**	22	4	Frontal_Inf_Tri_R (88%), Insula_R (12%)	33	6.07	5.01	0.37
**36**	-4	52	Precentral_R (57%), Frontal_Mid_2_R (33%), Frontal_Sup_2_R (10%)	21	6.05	4.90	0.42
**32**	18	4	Insula_R (61%), Pallidum_R (10%)	31	5.59	4.95	0.30
**-46**	52	2	Frontal_Sup_2_L (100%)	25	5.42	4.86	0.29

Table S1.4 : ***noFB* > *rest***

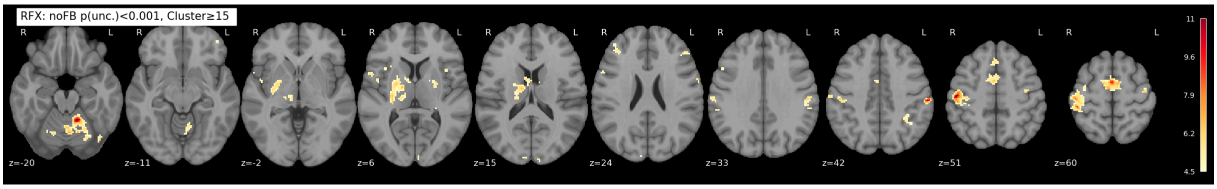

X	Y	Z	Region	Size	PeakT	MeanT	StdT
**-14**	-48	-22	Cerebellum_7b_L (44%), Cerebellum_6_L (38%), Cerebellum_Crus2_L (8%), Vermis_9 (5%)	780	22.60	6.01	1.92

**50**	-18	52	Parietal_Sup_R (64%), Precentral_R (31%)	872	14.64	5.65	1.21
**0**	-4	68	Supp_Motor_Area_R (55%), Rolandic_Oper_L (40%)	735	13.99	5.86	1.36
**14**	-10	20	Pallidum_R (17%), Heschl_R (12%), Thal_IL_R (8%), Putamen_R (8%), Thal_Re_R (6%)	789	13.31	5.33	0.89
**-60**	-24	42	SupraMarginal_L (69%), Parietal_Inf_L (20%), Postcentral_L (6%)	142	11.89	5.97	1.52
**18**	-64	-24	Cerebellum_8_R (94%)	230	9.45	5.37	0.82
**-26**	-34	10	Hippocampus_L (5%)	19	8.97	5.49	1.12
**24**	2	68	Frontal_Sup_2_R (100%)	38	8.20	5.46	0.95
**-64**	2	22	Postcentral_L (67%), Background (7%)	15	7.96	5.55	0.97
**-40**	-12	62	Background (97%)	58	6.98	5.37	0.65
**54**	10	28	Frontal_Inf_Oper_R (85%), Precentral_R (12%)	65	6.65	5.13	0.55
**48**	0	2	Insula_R (77%), Rolandic_Oper_R (23%)	31	6.48	5.15	0.56
**-22**	-18	20	Caudate_L (16%), Thal_VPL_L (10%)	50	6.33	4.98	0.39
**-8**	-98	14	Calcarine_L (41%), Cuneus_L (31%), Occipital_Sup_L (26%)	39	6.17	4.93	0.33
**10**	-96	20	Lingual_R (44%), Occipital_Mid_R (12%)	16	6.10	5.03	0.46
**56**	8	6	Rolandic_Oper_R (53%), Frontal_Inf_Oper_R (25%), Temporal_Pole_Mid_R (22%)	32	6.06	4.91	0.40
**42**	46	22	Frontal_Mid_2_R (100%)	73	5.92	4.94	0.34
**-32**	-46	40	Parietal_Inf_L (100%)	51	5.75	4.83	0.29
**-12**	-2	12	Putamen_L (52%), Thal_VL_L (15%), Caudate_L (6%)	62	5.74	4.84	0.27
**-44**	50	-14	OFCpost_L (53%), Frontal_Sup_2_L (29%), Frontal_Inf_Tri_L (6%)	17	5.53	4.92	0.27
**-40**	12	8	OFClat_L (95%), Frontal_Mid_2_L (5%)	19	5.31	4.80	0.24
**56**	-36	30	Angular_R (100%)	17	5.28	4.80	0.21
**38**	16	2	Insula_R (81%)	32	5.25	4.80	0.20
**34**	-10	58	Frontal_Sup_2_R (58%), Precentral_R (42%)	19	5.23	4.77	0.21
**-50**	36	24	Frontal_Inf_Oper_L (65%), Frontal_Sup_2_L (35%)	23	5.17	4.81	0.21

Table S1.5 : *cFB > aFB*

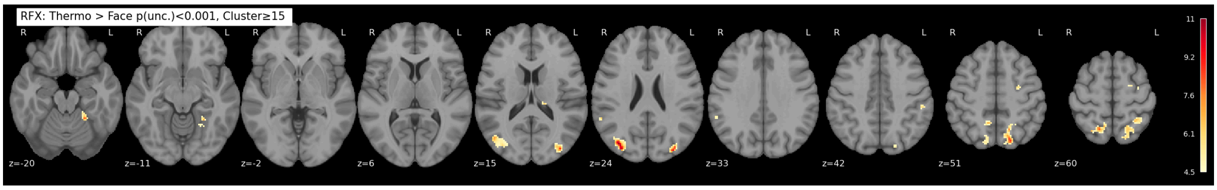

X	Y	Z	Region	Size	PeakT	MeanT	StdT
**-22**	-46	-18	Fusiform_L (47%), Cerebellum_6_L (37%), Lingual_L (15%)	113	17.34	5.80	1.70
**14**	-64	66	Parietal_Inf_R (51%), Paracentral_Lobule_R (48%)	225	14.69	5.75	1.35
**36**	-80	28	Occipital_Inf_R (78%), Temporal_Inf_R (17%)	464	13.70	5.96	1.53
**-30**	-86	28	Occipital_Mid_L (100%)	170	12.42	6.02	1.44
**-16**	-72	54	Parietal_Sup_L (75%), Precuneus_L (22%)	486	10.68	5.46	0.89
**-16**	-32	14	Thal_PuA_L (57%)	35	9.63	5.83	1.22
**-28**	-10	48	Background (77%), Precentral_L (10%)	61	9.54	5.51	0.96
**58**	-44	30	Angular_R (59%), Temporal_Mid_R (41%)	46	6.95	5.19	0.56
**-18**	-8	56	Precentral_L (46%)	28	6.87	5.17	0.50
**-16**	-68	-26	Cerebellum_7b_L (57%), Cerebellum_Crus2_L (43%)	23	6.42	5.30	0.49
**-56**	-28	36	Parietal_Inf_L (75%), SupraMarginal_L (25%)	28	6.08	5.11	0.44
**10**	-72	54	Paracentral_Lobule_R (53%), Parietal_Inf_R (47%)	34	5.86	5.01	0.35

Table S1.6 : *aFB > cFB*

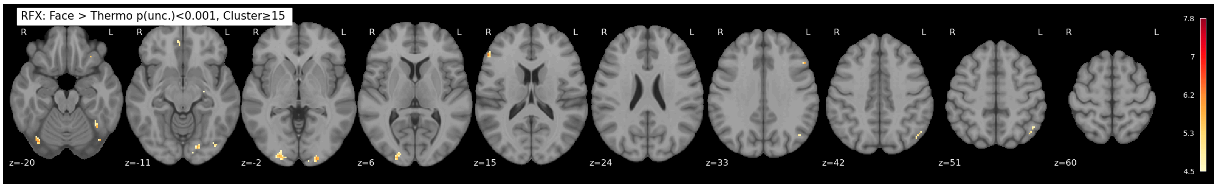

X	Y	Z	Region	Size	PeakT	MeanT	StdT
**-24**	-12	-16	Hippocampus_L (93%), Amygdala_L (7%)	15	11.46	5.56	1.72
**-20**	-84	-10	Occipital_Inf_L (29%), Calcarine_L (26%), Occipital_Mid_L (21%), Lingual_L (18%), Fusiform_L (6%)	171	8.56	5.35	0.79
**-32**	32	-16	OFCant_L (95%)	21	7.66	5.91	0.79
**-50**	22	32	Frontal_Mid_2_L (41%), Frontal_Inf_Oper_L (41%), Frontal_Sup_2_L (18%)	17	7.23	5.49	0.76
**36**	-76	-22	Cerebellum_3_R (42%), Cerebellum_8_R (35%), Postcentral_R (22%)	40	6.79	5.30	0.63
**-46**	38	20	Frontal_Sup_2_L (71%), Frontal_Inf_Oper_L (29%)	17	6.65	5.10	0.53

**54**	32	14	Frontal_Inf_Tri_R (100%)	31	6.34	5.20	0.47
**-40**	-76	-18	Fusiform_L (74%), Occipital_Inf_L (17%), Cerebellum_Crus2_L (6%)	35	6.32	5.02	0.45
**-50**	-60	48	Parietal_Inf_L (51%), Angular_L (46%)	37	6.22	5.17	0.43
**6**	48	-14	Frontal_Med_Orb_R (100%)	20	5.93	5.01	0.39
**24**	-94	-2	Cuneus_R (38%), Fusiform_R (25%), Occipital_Mid_R (21%), Occipital_Inf_R (8%), Occipital_Sup_R (7%)	72	5.88	4.99	0.36
**-50**	-66	40	Angular_L (95%)	40	5.78	5.04	0.36
**-38**	-58	-20	Fusiform_L (100%)	19	5.73	4.97	0.36

Table S1.7 : *noFB > FB*

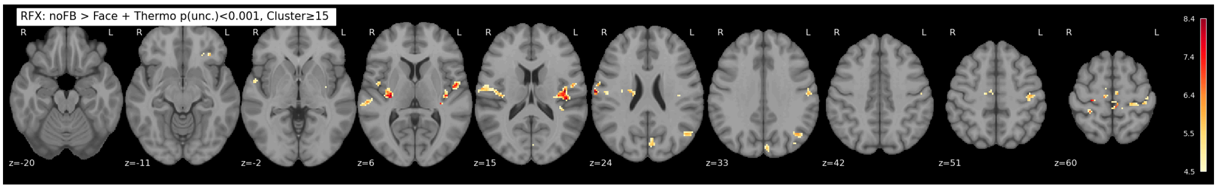

CLUSTER-REPORT: RFX: noFB > Face + Thermo

X	Y	Z	Region	Size	PeakT	MeanT	StdT
**64**	-14	24	Rolandic_Oper_R (41%), Insula_R (20%), Parietal_Sup_R (18%), Pallidum_R (8%)	391	12.13	5.50	1.04
**-42**	-12	14	Frontal_Inf_Orb_2_L (49%), OFClat_L (38%), Temporal_Sup_L (7%)	274	10.35	5.82	1.07
**-48**	-8	2	Frontal_Inf_Orb_2_L (37%), Postcentral_L (31%), Temporal_Pole_Sup_L (23%)	101	10.33	5.59	1.07
**22**	-16	26	Putamen_R (24%)	45	9.21	5.52	1.00
**16**	-50	74	Parietal_Sup_R (61%), Parietal_Inf_R (26%), Paracentral_Lobule_R (12%)	74	8.99	5.81	1.10
**26**	-26	62	Precentral_R (85%), Parietal_Sup_R (12%)	41	8.47	5.77	1.06
**-42**	-20	48	Postcentral_L (72%), Background (28%)	313	8.23	5.36	0.78
**56**	-16	0	Temporal_Mid_R (100%)	62	8.21	5.22	0.67
**-38**	0	8	OFClat_L (100%)	21	8.20	5.41	0.85
**8**	-10	58	Supp_Motor_Area_R (100%)	60	8.17	5.29	0.70
**30**	-40	64	Parietal_Sup_R (100%)	30	8.16	5.42	0.88
**-6**	-34	66	Paracentral_Lobule_L (74%), Caudate_R (14%)	226	8.02	5.47	0.80
**-32**	34	-14	OFCant_L (64%), Frontal_Inf_Tri_L (24%), OFCmed_L (12%)	25	7.33	5.55	0.86
**-48**	-66	26	Angular_L (81%), Temporal_Pole_Mid_L (19%)	140	6.78	5.24	0.57
**-8**	-16	44	Cingulate_Mid_L (97%)	31	6.70	5.05	0.44
**-40**	-36	10	Temporal_Pole_Sup_L (56%), Frontal_Inf_Orb_2_L (15%), Temporal_Sup_L (15%)	48	6.52	5.23	0.54
**-6**	-78	26	Cuneus_L (100%)	87	6.49	5.07	0.44
**-6**	-14	62	Rolandic_Oper_L (79%), Paracentral_Lobule_L (21%)	28	6.23	4.99	0.49
**54**	0	-4	Temporal_Mid_R (77%), Temporal_Pole_Mid_R (23%)	26	6.07	5.05	0.43
**10**	-80	28	Lingual_R (100%)	22	5.25	4.80	0.23
**-42**	-78	30	Occipital_Mid_L (94%), Angular_L (6%)	16	5.21	4.89	0.21
**-6**	56	-8	Frontal_Sup_Medial_L (100%)	19	5.15	4.77	0.15
